# User-Centered Development and Testing of the Online Patient-Reported Outcomes, Burdens, and Experiences (PROBE) Survey and the myPROBE App and Integration With the Canadian Bleeding Disorder Registry: Mixed Methods Study

**DOI:** 10.2196/30797

**Published:** 2022-03-02

**Authors:** Federico Germini, Victoria Borg Debono, David Page, Victoria Zuk, Alexandra Kucher, Chris Cotoi, Nicholas Hobson, Michael Sevestre, Mark W Skinner, Alfonso Iorio, PROBE Investigators

**Affiliations:** 1 Department of Health Research Methods, Evidence, and Impact McMaster University Hamilton, ON Canada; 2 Department of Medicine McMaster University Hamilton, ON Canada; 3 Canadian Hemophilia Society Montreal, QC Canada; 4 Patient Outcomes Research Group Ltd Washington, DC United States; 5 Design2Code Inc Waterloo, ON Canada; 6 Institute for Policy Advancement Ltd Washington, DC United States; 7 see Acknowledgments Hamilton, ON Canada

**Keywords:** health-related quality of life, EQ-5D, mobile app, Patient-Reported Outcomes, Burdens, and Experiences (PROBE), hemophilia, mobile health, mHealth, eHealth, telehealth, user-centered design

## Abstract

**Background:**

The Patient-Reported Outcomes, Burdens, and Experiences (PROBE) questionnaire is a tool for assessing the quality of life and disease burden in people living with hemophilia.

**Objective:**

The objectives of our study were (1) to assess the needs of relevant stakeholders involved in the use of PROBE, (2) to develop the software infrastructure needed to meet these needs, and (3) to test the usability of the final product.

**Methods:**

We conducted a series of semistructured interviews of relevant stakeholders, including PROBE investigators, people with hemophilia, and representatives of the sponsor. Based on these, we developed an online survey and a mobile app for iOS and Android. A user group evaluated the final product using the System Usability Scale (SUS) and an open feedback framework.

**Results:**

The online survey was updated, and the myPROBE app for mobile devices and a new application programming interface were developed. The app was tested and modified according to user feedback over multiple cycles. The final version of the app was released in July 2019. Seventeen users aged 23 to 67 years evaluated the final version of the app using the SUS. The median (first, third quartile) SUS score for the app was 85 (68, 88) out of 100. The newly introduced functionalities were as follows: (1) capability to longitudinally track repeated fillings of the questionnaire at different time points by the same participant (as opposed to anonymous completion); (2) linking of the questionnaire with hemophilia registries, starting with the Canadian Bleeding Disorders Registry as a proof of concept; (3) removing or adding questions as needed; and (4) sending notifications to the users (eg, reminders). A new secure database was built for securely storing personal information separately from the questionnaire data. The PROBE online survey is currently available in 96 countries and 34 languages.

**Conclusions:**

The online survey was updated successfully, and the myPROBE app was developed, with a SUS score of 85 (out of 100). The app has been released in 81 countries and 34 languages. This will facilitate data collection for research and advocacy purposes, and the use of this tool in everyday clinical practice.

## Introduction

### Background

#### What is Hemophilia

Hemophilia is an inherited X-linked bleeding disorder. Hemophilia A is characterized by a deficiency in the clotting factor VIII, while Hemophilia B is a deficiency in factor IX. Given the reduced ability to form clots, people living with hemophilia experience an increased frequency and duration of bleeding events, which tend to occur mostly within the joints or muscles [1]. The standard of care for hemophilia treatment involves infusing factor replacement to increase factor concentrations in blood [1]. Another option for prophylaxis in people living with hemophilia A is subcutaneous infusions of emicizumab, a bispecific antibody that mimics the function of factor VIII [2].

#### Quality of Life in Hemophilia

Limiting the effects of bleeding episodes is important for maintaining patient quality of life, as bleeds cause deterioration of the joints and can result in pain and disability [3]. The quality of life of people living with hemophilia and the burden of the disease are also affected by other aspects, for example, the burden of adhering to a regular prophylactic regimen and comorbidities, particularly bleeding-related arthropathy and transfusion-transmitted infections with hepatitis B virus, hepatitis C virus, or HIV [4]. Outcome assessment in hemophilia often comprises clinical measures, such as number of target joints, number of emergency room visits, and hospital length of stay [5]. In recent years, patient-reported outcomes (PROs) have been increasingly used to measure additional metrics that capture a patient’s perspective of their own health [6,7]. The assessment of PROs involves asking an individual to assess a variety of domains, such as pain, disability, function, and satisfaction with treatment. This, when combined with clinical outcomes, provides a more comprehensive understanding of the disease and available treatments [8]. Multiple PROs and quality of life measuring instruments exist in hemophilia, although they have not involved patients throughout the development of the questionnaire, despite clear guidance to do so [9]. The Patient-Reported Outcomes, Burdens, and Experiences (PROBE) questionnaire was developed for patients by patients, with the support of an epidemiologist, an expert in outcome research, an expert in management science, and economics-engineering systems [10]. The PROBE questionnaire has undergone assessment for feasibility, validity [10], and test-retest reliability (including cross-validation of paper and online versions) [11], and evaluation of other psychometric properties [12]. The finalized version of the PROBE questionnaire is composed of 29 questions with additional subquestions depending on responses, which have been divided into 4 sections, focusing on personal demographics, general health problems, disease-specific health problems, and the EuroQol 5 dimensions 5 levels (EQ-5D-5L) and Visual Analog Scale (VAS). Before this project, PROBE had been tested across 21 countries, and was available in 11 languages (with 20 localized versions worldwide). It has been shown that PROBE is a valid questionnaire for the evaluation of PROs in people living with hemophilia and a control population [12]. Its discriminative properties allow its use in clinical trials, longitudinal studies, health technology assessment studies, routine clinical care, or disease registries. Before this project, the questionnaire could be filled on the PROBE website anonymously by people living with hemophilia and controls (individuals without a bleeding disorder) alike. This system did not allow for longitudinal data collection. If the same individual returned at a later date to take the questionnaire again, the new questionnaire was not linked to the previous one. The PROBE investigators assessed that the capacity to collect longitudinal data and to link responses to other databases would provide benefits to physicians, people living with hemophilia, and researchers. By doing so, if agreed by the participant, individual responses could be tracked, and progress could be monitored and evaluated.

#### Digital Health Interventions for Chronic Conditions

The acceptability and feasibility of digital health interventions in chronic conditions (including rare diseases) have already been proven, while evidence about the efficacy of such interventions on patient-oriented outcomes is not definitive [13-16]. In our case, we aimed at eliciting the measurement of PROs. This is not an intervention directly aimed at affecting outcomes, even though this can be an indirect effect. Longitudinal PRO data could support decisions surrounding novel treatments or treatment schedule changes, provide important insights on the changes in quality of life following certain events (eg, bleeds, surgery, or a change in treatment regimen), or provide a tool for physicians or health systems to track patient outcomes over time. Different solutions have been proposed for the digital collection of PRO measures, including modifying the architecture of the electronic health record to integrate data collection from different mobile apps [17]. However, once implemented, the usability of these systems needs to be tested, as changes might be needed if the results are unsatisfactory [18,19].

The backbone of this project was making prospective longitudinal data collection of the PROBE questionnaire possible, by developing a stand-alone individual longitudinal PROBE modality and integrating the data collection of PROBE with other databases. The Canadian Bleeding Disorders Registry (CBDR) was used to demonstrate the feasibility of registry integration, and the usability of the app was tested.

### Objectives

The objectives of the project were as follows: (1) to assess the needs of the relevant involved stakeholders, (2) to develop the software infrastructure needed to meet those needs, and (3) to test the usability of the final product with potential users.

## Methods

### Study Phases

The study was organized in the following phases: (1) needs assessment, (2) identification of software specifications, software development, and beta testing, and (3) usability test of the final product.

#### Needs Assessment

To plan for the development of the software infrastructure and design of the PROBE project, we conducted a series of semistructured interviews with a convenience sample of front-end and back-end users, including all relevant stakeholders. In detail, back-end users were the PROBE investigators, representatives of the sponsor, and researchers, and front-end users were people with hemophilia. The interviewees were based in Canada, Ireland, Italy, Switzerland, and the United States of America. In preparation for the interview, we asked the interviewees to complete the PROBE questionnaire on the website, unless they had recently taken it. The semistructured interview guide is provided in Textbox 1. Characteristics of the stakeholders are provided in Multimedia Appendix 1.

**Guide of the semistructured interview for the needs assessment phase.**

1. What’s your role in Patient-Reported Outcomes, Burdens, and Experiences (PROBE), if any?

2. What do you like of the current PROBE website?

3. What is that you do not like of it?

4. What functionalities do you think are missing in the PROBE website/paper version?

5. What future do you envision for PROBE, especially in terms of its use for advocacy, clinical research, and clinical activity?

6. Do you think updating the website would be enough for these scopes, or a mobile app is needed?

#### Software Specifications, Development, and Beta Testing

We put together a technical group involving (1) 3 programmers on staff at the Health Information Research Unit at McMaster University, (2) an external consultant (Design2Code [D2C]) based in Waterloo and skilled in mobile app development, and (3) stakeholder representatives. The needs identified in the previous phase were used to guide the creation of 2 alternative plans for software development: updating the website and creating a mobile app or only updating the website to meet those needs. The 2 plans were discussed within the technical group using the Strengths, Weaknesses, Opportunities, and Threats (SWOT) analysis process to compare the different alternatives, including their costs and the time needed for development. The product of this phase was a justification of the choice and a detailed description of the technical specifications for realizing the PROBE suite. The main areas of work were defining if an app was needed and which characteristics were required, and understanding how the existing PROBE website, database, and application programming interface (API) needed to be modified.

All members of the technical team and selected PROBE investigators were invited to test the software under development and provide feedback. Testing included 3 sessions of group testing and independent individual testing. The first round of testing and feedback was based on a mock-up of the product. The second round was based on the beta version of the new website and the app, released on TestFlight for iOS and on the testing environment of Google Play. These products were working with a fake development environment (API and database). The third round was based on the first release of the website and mobile app, using a test modality on the production database.

#### Usability Testing

Once the software was stable in its first version, which took 3 releases, formal usability testing was performed. A user group composed of a convenience sample of patients, representatives of the sponsor from the same countries specified above, and graduate students from McMaster University evaluated the final product with the System Usability Scale (SUS) [20] and an open feedback framework. The SUS is composed of 10 questions (reported in Multimedia Appendix 2) to be answered on a 5-point Likert scale, from “strongly agree” to “strongly disagree.”

### Statistical Analysis

The SUS score can range from 0 (worst usability possible) to 100 (best usability possible), calculated as suggested by John Brooke as follows: “to calculate the SUS score, first sum the score contributions from each item. Each item’s score contribution will range from 0 to 4. For items 1, 3, 5, 7, and 9, the score contribution is the scale position minus 1. For items 2, 4, 6, 8, and 10, the contribution is 5 minus the scale position. Multiply the sum of the scores by 2.5 to obtain the overall value of the SUS” [20]. The quantitative results of phase 3 of the project were presented using measures of central tendency and dispersion, or counts (frequencies) as appropriate. Data were analyzed using STATA/SE V.16.0 (StataCorp).

### Ethical Approval

The PROBE project was approved by the Hamilton Integrated Research Ethics Board (HIREB; application number 7492).

## Results

### Overview

The project started in November 2017. The needs assessment phase lasted 5 months. The project development phase started in March 2018. The usability test started in March 2019. The myPROBE app was officially released in July 2019.

### Needs Assessment

As a result of the semistructured interviews, a complete list of required functionalities was compiled. These functionalities are reported with the respective explanations in Textbox 2. The main needs that were identified included the longitudinal repetition of the questionnaire, linkage with other databases (starting with the CBDR as a proof of concept), and flexibility to allow adding or removing questions. There was a general agreement that these key functionalities were essential for the uptake of PROBE in clinical activity and clinical studies. Moreover, from the perspective of users, the possibility to complete the questionnaire on portable devices (smartphones and tablets) and to save an incomplete questionnaire and complete it later were identified as important features.

**List of functionalities for the new Patient-Reported Outcomes, Burdens, and Experiences (PROBE) suite.**

**Longitudinal repetition of the questionnaire**

Being able to identify two or more sets of answers to the Patient-Reported Outcomes, Burdens, and Experiences (PROBE) questionnaire as coming from the same user. This is key for the use of the questionnaire in clinical activities, in clinical studies, and to assess the responsiveness of the PROBE tool.

How the goal was achieved: Users are now asked to create a username and a password and to login to the system before completing the questionnaire.

**Linking PROBE data with other databases**

Many registries on patients with bleeding disorders are available around the world. Linking these databases with PROBE data would allow using PROBE in clinical activity and enhance its use in research. Moreover, an increasing number of clinical trials are using PROBE to assess the efficacy of treatments.

The Canadian registry (Canadian Bleeding Disorders Registry [CBDR]) was selected to demonstrate proof of concept, leaving open the possibility to later add other registries and study databases in the future.

How the goal was achieved: Single sign-on with OAuth 2.0 technologies.

**Turning modules on and off**

When specific information is already available in a linked database or form previous questionnaires, it is not efficient to ask again, so some questions could be removed. If additional information must be collected for a specific study, some questions could be added.

How the goal was achieved: A survey manager allows the creation of new sections and questions for the PROBE questionnaire. A template builder allows to group sections and questions to generate different questionnaire templates.

**Completing the questionnaire on a portable device**

Smartphones and tablets are being more commonly used and are preferred to laptops and personal computers from many users. Moreover, smartphones are the only available devices to access the internet for the majority of users in low-income countries.

How the goal was achieved: A mobile app for iOS and Android was created.

**Saving an incomplete questionnaire and completing it later**

To minimize the loss of data and enhance the user experience.

How the goal was achieved: Data are saved locally (and submitted to the database, if a connection is available) every time a user answers a question.

**Send notifications to a patient**

For example, when it is time to repeat the questionnaire (eg, after 1 year, or after a bleed or an invasive procedure has been recorded in a linked database).

How the goal was achieved: For now, only email notifications can be sent to the users.

**Ensuring continuity of data collection**

The data from the PROBE questionnaire need to keep flowing to the existing PROBE database.

How the goal was achieved: Anonymized data are stored in the PROBE database, and personal identifiers are stored separately.

**Recording the time spent completing the questionnaire**

This was, for the PROBE investigators, an important measure of the questionnaire’s feasibility.

How the goal was achieved: The time elapsed from the questionnaire loading to its submission is recorded, and the information is stored in the database.

**Recording the questionnaire completion rate**

Again, to assess the feasibility of the questionnaire, the PROBE investigators need to track the number of users starting the questionnaire and the number of users submitting it in general and among users asked to complete the questionnaire via notifications.

How the goal was achieved: Every time a questionnaire is started, the answers are stored in the database. A variable identifies the questionnaires that have been submitted.

### Software Specifications and Development

#### App Versus Device-Responsive Website

Based on the results of the needs assessment and following the structured approach described as module 2 of the study, the PROBE team met with D2C to decide if an app was needed or if a device-responsive website was sufficient to achieve the objectives. The SWAT analysis results between the 3 plans were translated in pros and cons of having an app on top of the website, which have been summarized in Table 1. The team determined that an app was needed, primarily to facilitate the accessibility of the content, to send notifications to users when it is time to repeat the questionnaire (eg, after 6 months or when a bleed occurs), and to allow, in the future, leveraging of smartphone features like physical activity tracking or access to the camera. Furthermore, off-line questionnaire completion would not be possible on a website, while this was perceived as important, especially from the perspective of users from less developed countries where internet access or bandwidth is limited. Therefore, to meet these needs, it was agreed that an app for Android and iOS environments was required.

**Table 1:** Pros and cons of a mobile app and a device-responsive website.

Feature	App pro/con	Website pro/con
Accessibility from a portable device	Easily accessible through an icon.	Requires the user to save the bookmark on the home screen.
Notifications (eg, to repeat the questionnaire)	Available also while not using the app.	Only available while using the website or though emails/text messages.
Offline questionnaire completion	Connection required only to download and submit the questionnaire. Completion can happen offline.	Not accessible offline.
Leveraging smartphone features (eg, step count or camera)	Easier to achieve.	Harder to achieve.
General accessibility	Only accessible through a portable device. Specific operating system versions needed (eg, iOS and Android).	Accessible through a browser from a computer and a portable device.
Costs for development, maintenance, and update	In our case, a website was needed, so costs for the app would be added on top of website expenses.	Not having a website was not an option for the investigators.

#### Log-in Flow

To allow for the longitudinal repetition of the PROBE questionnaire, log-in options were required. Retaining the anonymous completion option was deemed important, as was adding an email-based login and a single sign-on (SSO) option for people living with hemophilia to participate through their national registry patient app (eg, myCBDR). The CBDR option was only made available to users selecting “Canada” as their home country.

#### Database Structure

**Figure 1:**
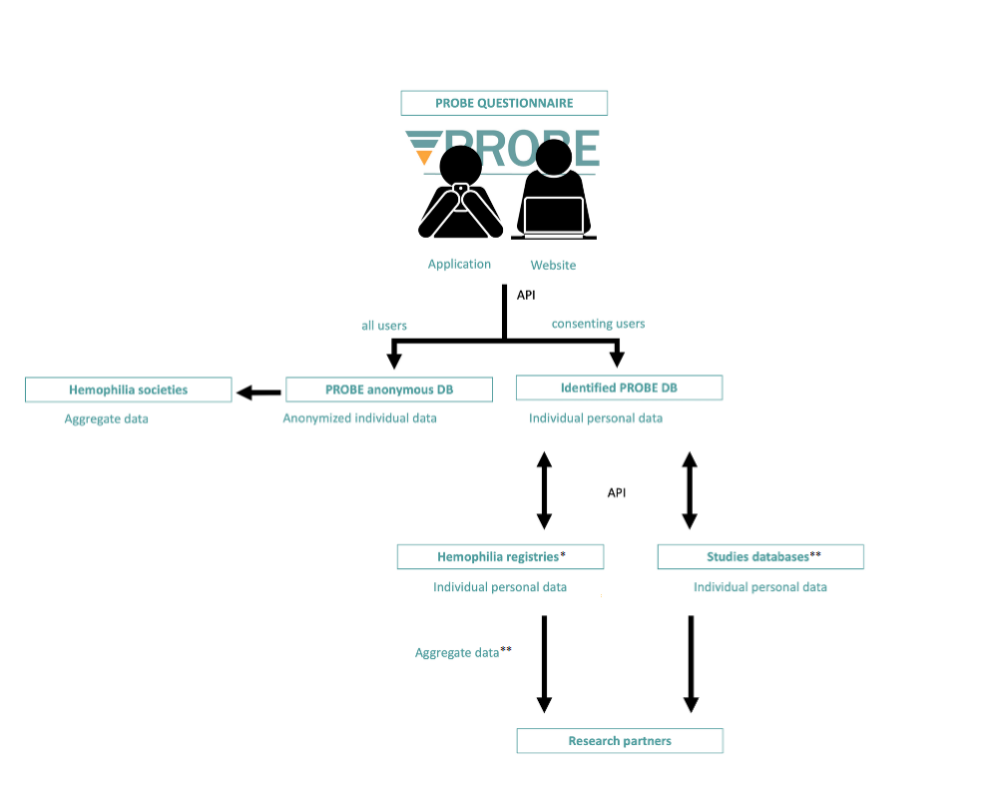
PROBE data flow. The pictures “using smartphone” and “using laptop” are by Llisole from the Noun Project (https://thenounproject.com). API: application programming interface; DB: database; PROBE: Patient Reported Outcomes, Burdens and Experiences. *Starting with the Canadian Bleeding Disorders Registry, with other registries in the future, **Based on future agreements.

The data flow from completion to data analysis and study reports is described in Figure 1. Individual data needed to flow from the app and the website to the PROBE database. For security reasons, personal information (email address or CBDR identifier) had to be stored separately from the PROBE questionnaire’s data. The capability to allow part of the data to flow bidirectionally from the PROBE database to others, like hemophilia registries, was needed.

#### API

Before this project, the PROBE website was communicating directly with the PROBE database. To implement the new functionalities, an API was required. Having an API in place allows (1) sending data to the databases from both the app and the website, (2) authenticating users with a dedicated email and password or with a token (eg, obtained using CBDR credentials or coming back to the PROBE website after the first log-in), and (3) supporting different versions of the questionnaire and other data (eg, notifications or calculation and report of PROBE and EQ-5D scores) from the database to the app and website. The technical specifications for the PROBE suite are reported in Multimedia Appendix 3 and Multimedia Appendix 4.

In collaboration with the PROBE investigators, the team at McMaster University and D2C developed an online survey using Microsoft.Net technologies and an app for iOS and Android using react-native. For the duration of the development phase, the McMaster University team and D2C met monthly to discuss progress and to find solutions to unanticipated problems. The PROBE investigators and the sponsor were involved as needed. Textbox 2 reports the solutions implemented to realize the main system functionalities. A sign-in and log-in interface was created. The SSO with MyCBDR credentials was implemented using OAuth 2.0 technologies. A survey manager and a template builder were implemented to allow creating new versions of the PROBE questionnaire by adding or removing sections and questions.

The 3 cycles of testing of the app and website allowed bugs and required fixes to be identified. Feedback from beta-testers led to improvements in the user experience, for example, adding autoscroll for pages displaying more than one question, changing the app buttons and graphics when they were perceived as not being clear, and adding descriptive text (eg, feet, inches, and pounds to the height and weight question for countries not using the metric system).

### Usability Testing

**Figure 2:**
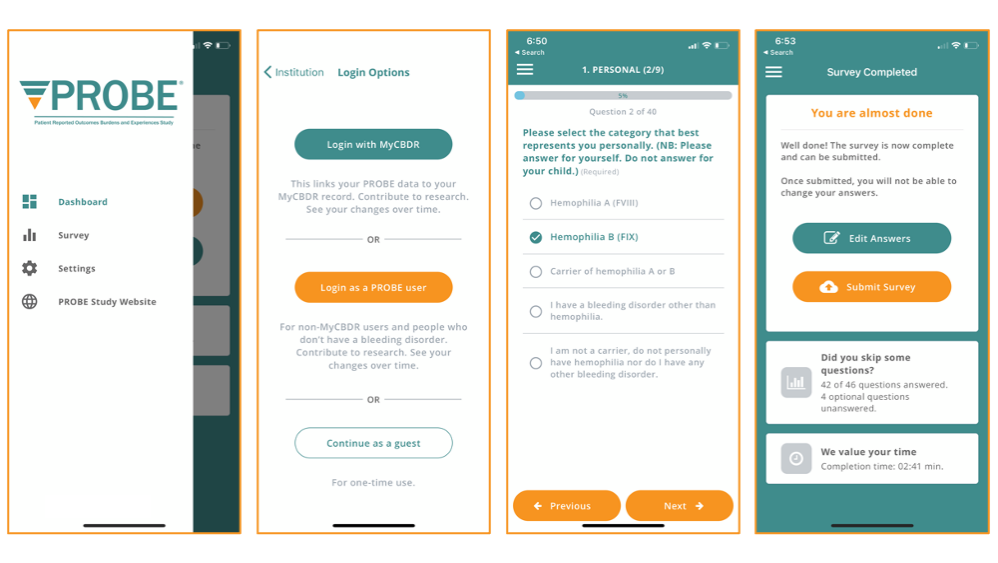
Screenshots of the myPROBE app.

Once a stable version was achieved, 17 users aged 23 to 67 years evaluated the app using the SUS. The median (first, third quartile) SUS score for the app was 85 (68, 88) out of 100. Based on the open-ended comments, most users indicated satisfaction. The only major edit requested was to allow data validation when possible. The app was modified accordingly (eg, “age” was restricted to be a number between 0 and 100). Figure 2 shows screenshots from the first release of the myPROBE app.

## Discussion

### Principal Findings

With a user-centered approach, careful needs assessment, and testing, a user-friendly mobile app was developed that allows longitudinal completion of the PROBE questionnaire, administration of different questionnaires created ad hoc, and linkage with other databases through SSO. The myPROBE app was released on the Apple Store and Google Play Store in 81 countries and 34 languages. Testers favorably rated the usability of the app with a median score of 85 (out of 100).

### Strengths and Limitations

Time and resources for this project were limited. Therefore, some functionalities that users requested could not be implemented. We were successful in implementing SSO through MyCBDR, and recently added SSO with the Mexican Registry of Bleeding Disorders. However, for now, we are not supporting SSO with credentials from other services, like Apple or Google. Implementing this option might have relieved users from having to remember a new username and password specifically for PROBE, further increasing the usability of the app. Caregivers and researchers have a growing interest in linking quality of life (QoL) data with data on physical activity in people living with hemophilia. Having a mobile app paves the way for passive data collection through a wearable device (like a smartwatch). However, this is not yet possible for the myPROBE app. Some functionalities offered on the myPROBE website are not offered on the myPROBE app. In particular, the website allows users to download and share their questionnaire results and offers better support for participation in studies, with the possibility of administering different questionnaires to specific users. Additionally, functionalities have been recently developed but have not, as yet, been implemented on the myPROBE app. Retrocompatibility was limited to Android 5+ and iOS 10+. This might restrict access to the app, especially in low-income countries where older mobile devices may be commonly used. The strengths of our study include the multidisciplinary nature of the team, which involved experts in information technology and health research methodology, hemophilia treaters, people working in the industry, and, perhaps most importantly, people with hemophilia. We believe that the user-centered approach with early involvement of final users in the development of our product was key to determine the good usability of the app. The widespread distribution of the app and its translation in more than 30 languages will favor its uptake and will foster new feedback on how to further improve it.

### Comparison With Prior Work

To the best of our knowledge, myPROBE is the only mobile app for completing a QoL tool currently available on the market for people living with hemophilia.

The SUS has been widely up-taken by academics (6932 citations in Google Scholar on February 12, 2019) and practitioners, and it has been used in a large variety of settings, ranging from safety signs [21] to websites [22]. The SUS showed good to excellent psychometric properties in different settings [23]. Comparing the scores with benchmark measures [23], the myPROBE app was rated A+ (96th to 100th percentile).

In terms of mobile apps for collecting PRO data in patients with chronic conditions other than hemophilia, previous studies reported conflicting results. Welbie et al tested the usability of an app for PROs in physical therapy patients and found that users where overall satisfied with the usability of the app, but the app required some changes in navigation through questions and how to insert and edit answers [19]. The authors acknowledged that they would again test the usability of the app after implementing such changes. We found similar issues during our testing phase, and our approach was to implement such changes before testing usability. This confirms how it is critical to include direct patients’ inputs in the development of digital health interventions [13,24], and how key considerations for end users should be sought early on in the process of app or digital health intervention design to ensure short- and long-term engagement [25]. For example, it was shown how aspects that might be considered less important by researchers, like design, communication style, and user ratings, can be important for engagement [26]. Steele Gray et al investigated the usability of an app to report PROs in complex chronic disease and disability [18]. They highlighted issues with usability related to the frequency of questionnaire administration and actual use of the data in a clinical setting. The former issue is not directly related to the usability of a mobile app but more to the PRO tool in general. The former is an aspect that we did not explore in this study, as we wanted to focus on the usability of the app to collect data before moving to the use of the data in a clinical setting. Both the studies used qualitative approaches to explore the usability of the apps. On one side, this is valuable and allows more in-depth feedback and exploration of aspects that are not measurable. On the other hand, using a quantitative tool allows quantifying the usability and performing comparisons with other tools. For these reasons, we opted for a mixed methods approach, with a qualitative approach for development and initial feedback, and a quantitative approach for obtaining a measure of usability.

### Future Directions

We are continually expanding the global reach of PROBE with new releases of the myPROBE app, including new translations, thus making the app available in more countries and affording the opportunity to support the integration with other bleeding disorder registries. A frequently asked questions (FAQs) handout was produced in multiple languages, describing the scope of the PROBE project and app functionalities (Multimedia Appendix 5).

An SSO with the World Federation of Hemophilia World Bleeding Disorders Registry is being developed as we write this paper. A study to prove the test-retest reliability of PROBE when administered through the mobile app and the website is in process. Other future directions for the app will be addressing the above-mentioned limitations. In particular, funding for supporting passive data collection for physical activity is being pursued. There is growing interest in how to use PROBE data in clinical activity. We are working on offering the possibility to provide caregivers of people living with hemophilia (when people living with hemophilia consent) access to PROBE data, and to compare individual data with group data from other users [27]. For example, people living with hemophilia or their caregivers might want to know how they compare to people in the same age range and country, with or without hemophilia. Use of the PROBE questionnaire and benchmark data sets might prompt reflections on current problems and goals of care, potentially improving the care of people living with hemophilia. The integration with bleeding disorder registries offers the possibility of shortening the questionnaire and avoiding asking for information already available (eg, year of birth). Moreover, it would be possible to use data from these registries to prompt event-based completion of the questionnaire, for example, after a bleeding event is registered by a user or after a significant change in treatment access or standard of care in a country. Notifications might also be used to remind the user when it is time to repeat the questionnaire (eg, every 6 months).

In general, we believe that future studies on digital health interventions should involve end users in the early development phases, to ensure good usability and engagement. Moreover, adopting a widespread tool to measure usability would allow comparisons between different digital health interventions. Once developed, the real-world use of these tools should be assessed in terms of usability in a clinic setting ideally to see how these interactions can translate into patient care changes and if this can also affect outcomes. Lastly, economic evaluations should be performed to support the use of digital health interventions, and this aspect has been rarely investigated to date [28,29].

### Conclusions

The PROBE online survey was updated successfully, and the myPROBE app was developed using a user-centered approach. This allows digital administration of the PROBE questionnaire and other questionnaires, and adoption of SSO for ease of use and linkage to other databases. In the first months after the product’s release in 81 countries and 34 languages, the responses from testers and users have been largely positive. The median SUS score (85/100) compares well with previously published benchmark measures. We believe that this is a crucial step toward facilitating the use of this PRO tool in research and everyday patient care. This will contribute to pursuing the objectives of the PROBE project, including building a robust evidence base for comparative effectiveness, outcome research, evidence-based decision making, and advocacy.

## Appendix

Multimedia Appendix 1 Characteristics of the interviewees for the needs assessment phase (semistructured interviews).

Multimedia Appendix 2 System usability scale.

Multimedia Appendix 3 Technical requirements specification for the Patient-Reported Outcomes, Burdens, and Experiences (PROBE) service (excluding the PROBE app).

Multimedia Appendix 4 Technical requirements specification for the Patient-Reported Outcomes, Burdens, and Experiences (PROBE) mobile app for iOS and Android.

Multimedia Appendix 5 myPROBE app handout.

## Abbreviations

API: application programming interface
CBDR: Canadian Bleeding Disorders Registry
D2C: Design2Code
PRO: patient-reported outcome
PROBE: Patient-Reported Outcomes, Burdens, and Experiences
SSO: single sign-on
SUS: System Usability Scale
