# Supplementary material for: User-Centered Development and Testing of the Online Patient-Reported Outcomes, Burdens, and Experiences (PROBE) Survey and the myPROBE App and Integration With the Canadian Bleeding Disorder Registry: Mixed Methods Study
Source: JMIR Hum Factors. 2022 Mar 2;9(1):e30797. doi: 10.2196/30797 (PMC8928049; doi:10.2196/30797)
Supplement: Multimedia Appendix 1 [file humanfactors_v9i1e30797_app1.docx]

**Multimedia Appendix 1.** Characteristics of the interviewees for the needs assessment phase (semistructured interviews).

| N | 19 |
| --- | --- |
| Median (min, max) age: | 43 (22, 61) |
| Male, n (%) | 13 (68%) |
| Role, n* |  |
| PROBE investigator | 10 |
| Person with hemophilia | 6 |
| Hemophlia treater | 4 |
| Researcher | 9 |
| Sponsor representative | 2 |

*one person could belong to more than one category

structured interviews with a convenience sample of front-end and back-end users, including relevant stakeholders. Back-end users: the PROBE investigators (n = 10), hemophilia treaters (2), representatives of the sponsors (2), researchers(9). Front user: people with hemophilia (5). The interviewees were based in Canada, Ireland, Italy, Switzerland, and the United States of America.
