# Supplementary material for: User-Centered Development and Testing of the Online Patient-Reported Outcomes, Burdens, and Experiences (PROBE) Survey and the myPROBE App and Integration With the Canadian Bleeding Disorder Registry: Mixed Methods Study
Source: JMIR Hum Factors. 2022 Mar 2;9(1):e30797. doi: 10.2196/30797 (PMC8928049; doi:10.2196/30797)
Supplement: Multimedia Appendix 2 [file humanfactors_v9i1e30797_app2.pdf]

sFigure 1: system usability scale

For each of the following statements, please mark one box that best describes your reactions to *myPROBE app* today.

	Strongly disagree				Strongly agree
1. I think that I would like to use *myPROBE app* frequently.	1	2	3	4	5
2. I found *myPROBE app* unnecessarily complex.	1	2	3	4	5
3. I thought *myPROBE app* was easy to use.	1	2	3	4	5
4. I think that I would need the support of a technical person to be able to use *myPROBE app*.	1	2	3	4	5
5. I found the various functions in *myPROBE app* were well integrated.	1	2	3	4	5
6. I thought there was too much inconsistency in *myPROBE app*.	1	2	3	4	5
7. I would imagine that most people would learn to use *myPROBE app* very quickly.	1	2	3	4	5
8. I found *myPROBE app* very cumbersome (awkward) to use.	1	2	3	4	5
9. I felt very confident using *myPROBE app*.	1	2	3	4	5
10. I needed to learn a lot of things before I could get going with *myPROBE app*.	1	2	3	4	5

Example of the system usability scale (SUS) for the myPROBE app, produced from the website https://www.usabilitest.com.
