# Supplementary material for: User-Centered Development and Testing of the Online Patient-Reported Outcomes, Burdens, and Experiences (PROBE) Survey and the myPROBE App and Integration With the Canadian Bleeding Disorder Registry: Mixed Methods Study
Source: JMIR Hum Factors. 2022 Mar 2;9(1):e30797. doi: 10.2196/30797 (PMC8928049; doi:10.2196/30797)
Supplement: Multimedia Appendix 3 [file humanfactors_v9i1e30797_app3.pdf]

Technical Requirements Specification for the PROBE service (excluding the PROBE App)

This document describes how the new PROBE-R (Research) infrastructure will be developed to integrate with the existing PROBE infrastructure, by the HIRU's IT staff, and allow the longitudinal data collection of the PROBE questionnaire as described in the main document. Technical specifications are discussed here for all the aspects of the project except for the mobile app. The mobile app will be outsourced, and technical specifications for that are provided in Appendix 2.

A graphical description of the overall infrastructure is provided in Figure A, with different colors indicating what already exists, what will be implemented by the HIRU's IT staff, what will be outsourced to Design2Code, and the services provided by other external entities.

Figure A: PROBE extension diagram

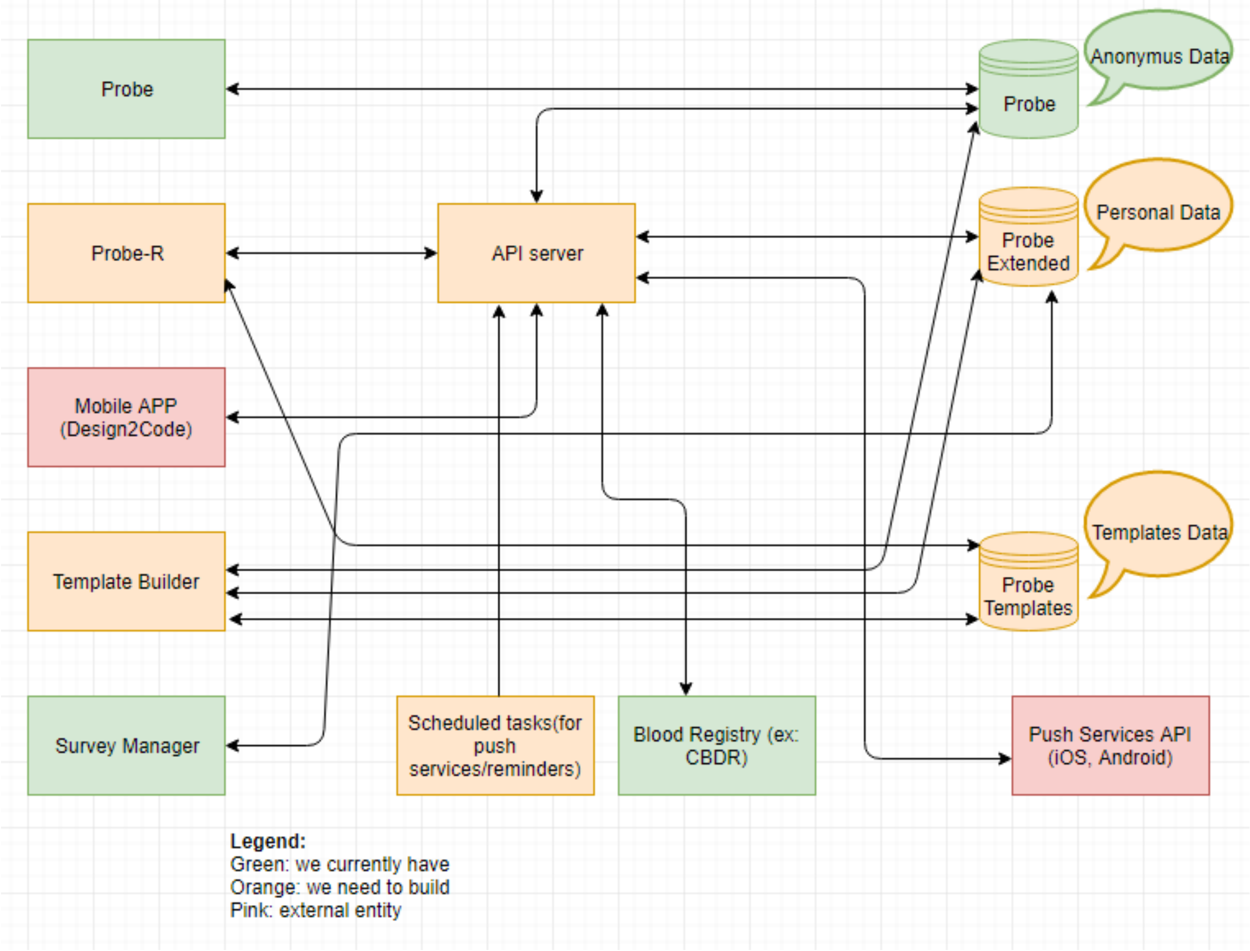

Detailed description of the components

PROBE-R website

This is a new website that will be created to allow the longitudinal repetition of the PROBE questionnaire. For this purpose, PROBE-R will allow the collection of users' identifiers, the integration with bleeding disorders registries (starting with the Canadian Bleeding Disorders Registry, CBDR) and the collection of data in the frame of clinical studies, with the possibility to customize the questionnaire.

Front-end functionalities

User interface for Probe-R

- Registration and authentication via email and password
- Authentication via unique token
- Registration and authentication via MyCBDR credentials (MyCBDR is the users' interface of MyCBDR)
- Registration and authentication via a machine generated PIN

Questionnaire Management

- Display the questionnaire
- Start the questionnaire
- Resume a questionnaire and edit if not submitted
- Navigate between questions

Multi-language Support

- Initially, the user interface will support English and French.
- The users will be able to choose from a list of languages that are already available.

Back-end capacities

User Settings

- Management of users' credentials
- Save country of origin
- Save preferred language
- Store information that is not likely to change over time (as specified in the main document), and re-propose them for submitting/editing in subsequent questionnaires.

Questionnaire Management

- Support for additional questions (to be added on top of the core PROBE questions)

Patient Notification

- Send email reminders for the longitudinal repetition of the questionnaire. Reminders will be based on the time elapsed from the last questionnaire. Also send reminders based on polling CBDR for occurrences of specific events (e.g. bleeding, invasive procedures). The latter option only applies to CBDR users, in the future might be extended to other countries' registries or clinical studies.

PROBE website

This is the already existing website. Users who choose to complete the questionnaire anonymously will be directed here from the PROBE-R website.

Application programming interface (API) Service

An API will be created, in order to allow:

- The mobile APP to exchange data with various systems (PROBE databases - DBs - , Registries - starting with CBDR -, Study DB in the future). Create all the methods necessary to accomplish the task
- The PROBE-R website to communicate with various registries, PROBE DB, and Probe Templates DB.

The API server will manage the following functionalities:

- Authenticate User using email and password
- Authenticate User using PIN
- Authenticate User using app Token
- Read from PROBE Template DB and serve the questionnaire template to the APP
- Read from PROBE Template DB and serve the questionnaire template to PROBE-R
- Accept data from the app, triage and save the data in PROBE and/or PROBE Extended DB all at once.
- Accept data from the PROBE-R, triage and save the data in PROBE and/or PROBE Extended DB

Survey Manager

The survey manager is a service that exists to facilitate modifying questions in the existing PROBE questionnaire and to generate questions to be added to the PROBE-R templates (e.g. if

this is needed for a new research project). In the first release, the survey manager will be available only on the back-end.

Template Builder

The Template Builder is a service that will be created to group sections and questions (generated through survey manager) to create PROBE-R templates(questionnaires).

It will allow:

- Creating new templates(questionnaires) using different sections/questions from PROBE and, optionally, additional questions (e.g. when needed for other projects).
- Retrieving questions and pre-requisites from other DBs.
- Generating new logical requisites for disparate survey questions (if needed).

PROBE DB (anonymous data)

This is the existing Data Base (DB). It contains the questionnaire answers and an ID which is unique for each questionnaire. This DB will remain as it is.

PROBE Extended DB (personal data)

PROBE Extended will hold the patient identifiers. This DB will be created to allow the longitudinal repetition of the questionnaire. For patients consenting to that, it will allow associating a user to the questionnaire answers and, when possible, to the users' CBDR profile.

PROBE template DB (templates/questionnaire data)

This DB will be created to store the different versions of the PROBE questionnaire and any other section or question created for future ancillary research projects.

Scheduled Tasks

This service will be created to facilitate generation of reminder emails to longitudinal participants, as well as to trigger Push Notifications to specific devices (for APP users) based on events as described above (currently only in CBDR).
