# Supplementary material for: User-Centered Development and Testing of the Online Patient-Reported Outcomes, Burdens, and Experiences (PROBE) Survey and the myPROBE App and Integration With the Canadian Bleeding Disorder Registry: Mixed Methods Study
Source: JMIR Hum Factors. 2022 Mar 2;9(1):e30797. doi: 10.2196/30797 (PMC8928049; doi:10.2196/30797)
Supplement: Multimedia Appendix 4 [file humanfactors_v9i1e30797_app4.pdf]

Technical Requirements Specification for the PROBE Mobile Application for iOS and Android

1 Introduction

1.1 Purpose

This document describes the mobile application **PROBE_APP** being developed for the realization of the level 2 PROBE service. The goal of **PROBE_APP** is to allow PROBE users to complete a PROBE questionnaire directly from their mobile device. The questionnaire should be available for offline use and authenticated users should be reminded to take upcoming questionnaires via notifications.

1.2 Product Scope

The PROBE service is already available today at https://plus.mcmaster.ca/PROBE. User data is collected anonymously and stored in the PROBE database. Although the PROBE website is already mobile responsive, a stable internet connection is required at all times in order to complete the survey. Poor or intermittent internet connectivity had lead in the past to data loss where users were required to take the questionnaire again.

The goal of **PROBE_APP** is first and foremost to allow the longitudinal data collection of PROBE questionnaires. This will be achieved by introducing an optional user authentication mechanism in **PROBE_APP**. Users will have the possibility to either login using their registry credentials if available (for example CBDR in Canada) or to create a new user profile (as described in the main document). Alternatively, an anonymous survey can be filled by the user if desired.

Another goal of this implementation is to solve the connectivity and perceived responsiveness issues. **PROBE_APP** will enhance the overall user experience by allowing users to take surveys even when an internet connection is not available. Users will also have the option to seamlessly interrupt the questionnaire and finish it at a later time.

2 Overall Description

2.1 Product Functions

PROBE_APP will contain the following hig level features:

User Authentication

- Select country of origin and language
- Registry authentication
- Sign up using email and password (using the email as username or using it to generate a new username)
- Login using username and password
- Automatic login if authentication token available

Questionnaire Management

- Download the questionnaire
- Start the questionnaire
- Start the questionnaire anonymously
- Resume an incomplete questionnaire
- Submit completed questionnaire
- Support for different question types
- Optional questions

- Navigation between questions

User Notification

- Display push notifications from PROBE Server / Service
- Display local notifications

Multi-language Support

- User interface will support a subset of all languages supported by PROBE
- Questionnaire will be displayed in the selected language

Offline Availability

- Questionnaire template will be saved in local storage
- Answers to current questionnaire will always be saved locally

User Settings

- Save country of origin and preferred language to device storage
- Save user preferences

2.2 User Classes and Characteristics

PROBE_APP will not offer role based features.

User:

- Can sign up to the PROBE Service
- Can log in
- Can take an anonymous survey
- Can take a new survey
- Can resume an existing incomplete survey
- Can submit a completed survey

- Can log out

2.3 Operating Environment

PROBE_APP will be available for Android and iOS. The supported platform version will be:

- For Android: Version 4.4 (KitKat) and above
- For iOS: Version 8 and above

2.4 Design and Implementation Constraints

PROBE_APP will communicate exclusively with the probe server via the PROBE API implemented by McMaster University.

3 System Features

3.1 User Authentication

REQ-3.1.1 Select country of origin and language

When **PROBE_APP** is starting for the first time or when users have logged out, a Startup Page should be displayed inviting the user to select their country of origin.

1. User starts the app for the first time or after logging out.
2. A request to retrieve the list of all available countries is sent to the PROBE API.
3. The PROBE API response contains the list of available countries.
4. Default selected country is set to the last selected country if available.
5. The language selection is populated with all languages supported by **PROBE_APP**.

REQ-3.1.2 Registry authentication

Users originating from a country with a registry supported by PROBE (Canada and CBDR, for this first release of the PROBE service), should be encouraged to log in using their registry credentials, if they are person leaving with hemophilia.

1. User selects a country with a supported Registry (e.g. Canada and CBDR).
2. User selects the language in which the survey should be taken.
3. The MyCBDR authentication mode is shown to the user.
4. User enters the registry credentials (e.g. MyCBDR username and password).
5. A login request with provided credentials is sent to the PROBE API.
6. The PROBE API response contains an authentication token for a successful authentication to be passed along as header to each subsequent request.
7. Token is saved on device local storage.
8. User is directed to the Home Page of **PROBE_APP**.

REQ-3.1.3 Sign up using email and password

Users residing in a country where no registry integration is available (or for which it is not possible to access through MyCBDR) should be able to sign up and create a profile using their email and a password.

1. User selects a country (without registry integration).
2. User selects the language in which the survey should be taken.
3. User clicks on the create account / register button.
4. User enters email and password.
5. A sign-up request with provided email and password is sent to the PROBE API.

6. The PROBE API response contains an authentication token for a successful authentication to be passed along as header to each subsequent request.
7. Token is saved on device local storage.
8. User is directed to the Home Page of **PROBE_APP**.

REQ-3.1.4 Login using email and password

A user residing in a country where no registry integration is available (or for which it is not possible to access through MyCBDR) should be able to login using email (or a username automatically generated using the email) and password

1. User selects a country without a supported registry.
2. User selects the language in which the survey should be taken.
3. User clicks on the login / sign in button.
4. User enters username and password.
5. A sign-in request with provided email and password is sent to the PROBE API.
6. The PROBE API response contains an authentication token for a successful authentication to be passed along as header to each subsequent request.
7. Token is saved on device local storage.
8. User is directed to the Home Page of **PROBE_APP**.

REQ-3.1.5 Automatic login if authentication token is available

To improve the overall user experience, users should not have to enter their login credentials every time **PROBE_APP** is started.

1. An existing user of **PROBE_APP** starts the application.
2. **PROBE_APP** checks if an authentication token is available in local storage.

3. If a token is available the user will be directed to the Home Page of **PROBE_APP**.
4. Otherwise the user will be redirected to the Startup Page of **PROBE_APP**.

3.2 Questionnaire Management

REQ-3.2.1 Download the questionnaire

The latest questionnaire will need to be downloaded and saved in **PROBE_APP** local storage based on the selected language. Download will be performed every time the user logs in and an internet connection is available to ensure that **PROBE_APP** is using the latest questionnaire available.

1. A request to retrieve the latest questionnaire is sent to the PROBE API for the selected language.
2. The response should contain the latest questionnaire in the selected language that will be saved in local storage for later use.

   Depending on the questionnaire size, we may decide to implement another strategy that would check first if the local questionnaire matches the latest questionnaire available online and only download the questionnaire if required.

REQ-3.2.2 Start the questionnaire

From the Home Page, the user will have the option to start a new questionnaire

1. The latest questionnaire is retrieved from local storage
2. The user is redirected to the Questionnaire Page
3. The first question is displayed to the user

REQ-3.2.3 Start the questionnaire anonymously

From the Login Page, the user will have the option to start a new questionnaire anonymously

1. The latest questionnaire is retrieved from local storage
2. The user is redirected to the Questionnaire Page
3. The first question is displayed to the user

REQ-3.2.4 Resume an incomplete questionnaire

The user should be able to resume a questionnaire already started. An incomplete questionnaire will only be available for two weeks after last save and will be discarded otherwise.

1. **PROBE_APP** checks during start/resume whether an incomplete questionnaire is available on the device.
2. If an incomplete questionnaire is found on the device, following cases will be taken into consideration:
    a) The time elapsed since last modification of the questionnaire is less than or equal to 2 weeks. The questionnaire is loaded and the last edited question is displayed to the user.
    b) The time elapsed since last modification of the questionnaire is more than two weeks. The questionnaire will be discarded and the user will be notified that the questionnaire is no longer available

REQ-3.2.5 Submit completed questionnaire

A completed questionnaire should be automatically submitted to PROBE upon completion. In the event that **PROBE_APP** is offline, or that the questionnaire submission was not successful, **PROBE_APP** will setup a local notification reminding the user to submit the questionnaire manually.

1. User completes the questionnaire
2. User submits completed questionnaire by clicking on the Submit button
3. A request is sent to the PROBE_API with the completed questionnaire
    a. If the PROBE_API responds with a success flag, the local questionnaire is deleted
    b. If the PROBE_API responds with an error flag, or if **PROBE_APP** is offline, a local notification will be created reminding the user to submit the questionnaire after a given period of time (for example one day). **PROBE_APP** can also try to submit a completed questionnaire automatically when started and online.

REQ-3.2.6 Support for different question types

The questionnaire will contain multiple questions. The type of the question will be defined in the question template. Following question types will be supported by **PROBE_APP**:

- Text Field
- Multiple choice
- Radio button (single choice)
- Range
- Date
- Numerical Field with units
- Table View.
    - This type of view does not work nicely with mobile resolution. This will be displayed as a list probably

REQ-3.2.7 Optional questions

The questionnaire may contain optional questions. The user should see a visual clue indicating whether the question is optional or compulsory.

1. The question to be displayed is loaded.
2. A marker indicating that the question is optional or compulsory is displayed to the user.

REQ-3.2.8 Navigation between questions

The user should be able to navigate between questions defined in the survey:

1. It should always be possible to navigate to the previous questions unless the current question is the first of the survey.
2. Navigation to next question should only be possible if the question is optional or the question was already answered by the user.
3. Moreover, each question comes with a dynamic criterion that will be parsed and evaluated to decide if moving to the next question is possible.
4. Navigation to the next question is not possible from the last question

3.3 User Notifications

REQ-3.3.1 Display push notifications from PROBE Server / Service

User should be notified when the time has come to fill the questionnaire again or upon trigger of certain events such as bleeds. A service triggering push notifications, either after a certain period of time or after some specific events needs to be implemented by McMaster with the help of Design2Code Inc. (see section Push Notification Server Support at McMaster)

1. Probe server sends notification to **PROBE_APP.**
2. Notification is being shown to the user if notification were authorized by user.

REQ-3.3.2 Display local notifications

If a survey was started but not completed, a local notification could be triggered after a given period of time (for example after a week) to remind the user that the survey was not finished. A notification could also be shown to the user if the survey was not synchronized with the server after a given period of time. This could happen if no internet connection was available while the app was up and running.

3.4 Multi-language Support

REQ-3.4.1 User interface will support a subset of all languages supported by PROBE

A Subset of all supported PROBE languages will be made available in the **PROBE_APP**.

User starts **PROBE_APP** for the first time or after logging out.

1. A language selection field is displayed on the Startup Page.
2. The default language will be specified as follow:
    a. If a previous language selection was available, select this language
    b. Otherwise select the default language for the selected country if available
    c. At last, select English as a default language

REQ-3.4.2 Questionnaire will be displayed in the selected language

The questionnaire should be displayed in the language selected by the user.

1. Upon selection of language, **PROBE_APP** sends a request to the PROBE_API to retrieve the latest questionnaire for the selected language.
2. The PROBE API response contains the questionnaire with all questions translated in the selected language.

3.5 Offline Availability

REQ-3.5.1 Questionnaire template to be saved in local storage

This is required to allow offline survey access. The latest questionnaire will always be downloaded when **PROBE_APP** starts and an internet connection is available.

REQ-3.5.2 Answers to current questionnaire will always be saved locally

Every time an answer is selected, it will be also saved locally. This will ensure that an interrupted survey can be resumed even if an internet connection is not available.

3.6 User Settings

REQ-3.6.1 Save country of origin and preferred language to device storage

The users should not have to select their country of origin and preferred language after each logout. This selection can be saved to device storage for later use.

REQ-3.6.2 Save user preferences

PROBE_APP will offer a user settings section with properties such as, but not limited to:

- Allow server notifications
- Allow local notifications

These properties will be saved on device local storage. Alternatively, these properties can also be saved in the profile database.

4 Other Non-Functional Requirements

4.1 Security Requirements

PROBE_APP will securely communicate with the PROBE server using the PROBE_API over HTTPS.

Each API calls to the PROBE API (except signup and login) should be authenticated. That means that an authentication token will be provided to each request to the API. The PROBE API will be responsible for checking the validity of this token.

PROBE_APP will not store any sensitive data (such as password) on the device.

PROBE_APP is not responsible for deciding where and how the data should be stored in the PROBE databases (anonymous or personal dataset). Instead **PROBE_APP** will only communicate to the PROBE service via the PROBE_API which will be responsible to manage and store the data according to PROBE service requirements.

4.2 Push Notification Server Support at McMaster

Push notifications are created from a service to specific devices / audiences / groups etc..

- They require an OS specific infrastructure that will send the notification on device
  - Apple Push Notification Service or APNS
  - Android Google Cloud Messaging or the new Firebase Push service
- Those services are not easy to work with. So generally, an app will register with those services but everything will be managed by a higher-level service. Let’s call this service **PUSH_SERVICE**, that will delegate to APNS and GCM for you. For example
  - OneSignal
  - Microsoft AppCenter (this is quite new and offers a lot of features)
  - Many more...
- It is possible to talk to **PUSH_SERVICE** via an API. This needs to be implemented on the server side.
- Some code will have to be run that checks when the survey should be taken again for a given user. If the times comes to take the survey again, the service will have to
  - Send an email if email is available

  - Send a push notification using the **PUSH_SERVICE** API to the given user

- Also push notification should be sent to ask a user to take a survey after a bleed was recorded in CBDR. When a bleed is recorded in CBDR, a push notification using the **PUSH_SREVICE** API will have to be sent informing the users that they should take the questionnaire again.
