# Supplementary material for: User-Centered Development and Testing of the Online Patient-Reported Outcomes, Burdens, and Experiences (PROBE) Survey and the myPROBE App and Integration With the Canadian Bleeding Disorder Registry: Mixed Methods Study
Source: JMIR Hum Factors. 2022 Mar 2;9(1):e30797. doi: 10.2196/30797 (PMC8928049; doi:10.2196/30797)
Supplement: Multimedia Appendix 5 [file humanfactors_v9i1e30797_app5.pdf]

myPROBE App handout

PROBE
Patient Reported Outcomes Burdens and Experiences Study

INTRODUCING

myPROBE App

Intuitive and Easy to Use

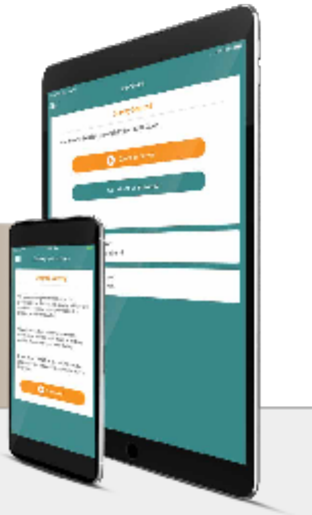

The myPROBE app allows respondents to complete the PROBE questionnaire on a smartphone or tablet.

What is PROBE?

PROBE stands for Patient Reported Outcomes Burdens and Experiences. PROBE is a long-term study aimed at measuring outcomes that people with hemophilia A and B think are important to their health status and quality of life. To gather data, people can complete this questionnaire via the myPROBE app.

Why use PROBE?

The PROBE study provides a way for people with hemophilia to report data on their health status and quality of life. Patient organizations can then use this data as evidence to advocate for better care and treatment.

Who can use PROBE?

The questionnaire can be completed by people with hemophilia A or B, including carriers. It can also be completed by people who don't have a bleeding disorder. They serve as the control group.

Get the App!

The app can be downloaded for FREE from the **Apple Store** for iOS and **Google Play** for Android and can be used on smartphones and tablets. Search for **myPROBE** in either the Apple Store or Google Play.

No device? No problem.
Complete the online survey at
myPROBE.org

Learn More Periodic updates, scientific abstracts and publications from the study are available on the PROBE website: **www.probestudy.org**

About PROBE

What does the myPROBE app questionnaire measure?

The myPROBE app questionnaire measures key outcomes such as pain, mobility, health impact on work and education, ability to conduct activities of daily living and quality of life.

How long does it take to complete the questionnaire?

Most people complete the questionnaire in less than 15 minutes.

How old does one have to be to complete the questionnaire?

There is no minimum age. One has to be old enough to understand the questions and provide answers. People with hemophilia as young as 11 have successfully completed the questionnaire. Parents must not complete the questionnaire for their children.

Who analyzes the data?

The PROBE investigators analyze the data collected. Only fully anonymized aggregated data will be reported. No identifiable information will be collected or disclosed. The data is stored on a secure server at McMaster University.

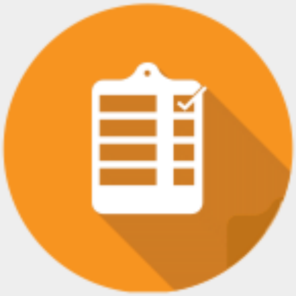

Complete the Questionnaire

There are 2 ways to complete the myPROBE questionnaire:

Option 1
The first way is to create a PROBE account, which allows the user to repeat the questionnaire in a year or two to compare results.

Option 2
The second way is to complete the questionnaire as a guest, which does not require login, but also does not allow individual data to be collected and compared over time.

Questions?

If you have questions or would like more information about PROBE, contact the PROBE investigator team at: **info@probestudy.org**

PROBE is a trademark of Patient Outcomes Research Group Ltd. **www.probestudy.org**
